# Infections of Deep Hand and Wrist Compartments

**DOI:** 10.3390/microorganisms8060838

**Published:** 2020-06-03

**Authors:** Konstantinos N. Malizos, Zoe K. Papadopoulou, Anna N. Ziogkou, Nikolaos Rigopoulos, Efstratios D. Athanaselis, Socrates E. Varitimidis, Zoe C. Dailiana

**Affiliations:** 1 Hand and Microsurgery Unit, Department of Orthopaedics and Musculoskeletal Trauma Medical School, University of Thessaly, PC41110 Biopolis-Larissa, Greece; anna_1908@hotmail.com (A.N.Z.); nirek@otenet.gr (N.R.); trts.a@gmail.com (E.D.A.); svaritimidis@hotmail.com (S.E.V.); dailiana@med.uth.gr (Z.C.D.); 2 2nd Surgical Department, G. Papanikolaou General Hospital, PC57010 Thessaloniki, Greece; zoetsa.papadopoulou@gmail.com

**Keywords:** hand infection, hand and wrist compartments, Parona’s space, septic arthritis, pyogenic flexor tenosynovitis, necrotizing fasciitis

## Abstract

The human hand is the most exposed part of the body to highest risk for injuries, loss of the skin integrity, and to the inoculation of bacteria, most commonly *Staphylococcus aureus*, *Streptococcus β-haemolytic*, and gram-negative. In case of an infection, the mobile anatomical structures and the synovial membranes in close proximity to each other may spread the pus towards deep spaces and compartments. Mild early infections without an abscess formation may respond to antibiotics, but at more advanced stage, erythema, swelling, stiffness, and severe pain may ensue. Abscess formation will cause debilitating pain, fever, systemic symptoms, and even sepsis. Necrotizing infections may threaten not only the limb, but also patient’s life. Therefore, an initially “trivial” hand injury should never be neglected, as it might turn into a deep space infection, which must be treated immediately with drainage, wound debridement, and i.v. antibiotics. Delay in diagnosis and inadequate initial management might rapidly lead to abscess formation, destruction of the gliding surfaces and the normal anatomy, and irreparable functional deterioration.

## 1. Introduction

Hand and wrist being the most exposed parts, are the most common injured sites of the human body. Even an apparently trivial and neglected wound caused by human or animal bites or been exposed to soil, still water, or contaminated surfaces may be inoculated with bacterial flora [1]. Subsequently, and depending on the virulence, anatomical location, local, and systemic host factors, might result in infections of variable severity. Despite modern antibiotics administration, these injuries continue to be a cause of morbidity and long-term disability [2,3]. 

## 2. Anatomical Compartments and Pathogenesis of the Deep Space Infections in the Hand

**Figure 1:**
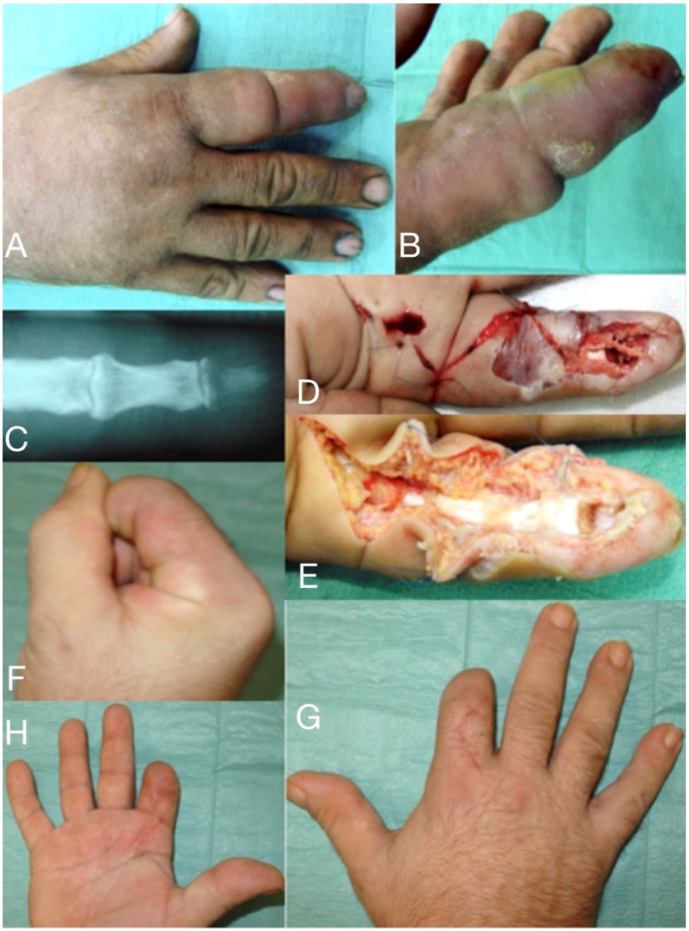
66-year-old male, seven days after a wood-thorn puncture in the index fingertip (**A**,**B**). Severe infection expanded to tenosynovitis, osteomyelitis of the distal phalanx (**C**), and septic arthritis of DIP joint. After debridement (**D**,**E**) viable tissue was left only in the proximal half of the index, which has been amputated (**F**–**H**) distal to the PIP joint.

**Figure 2:**
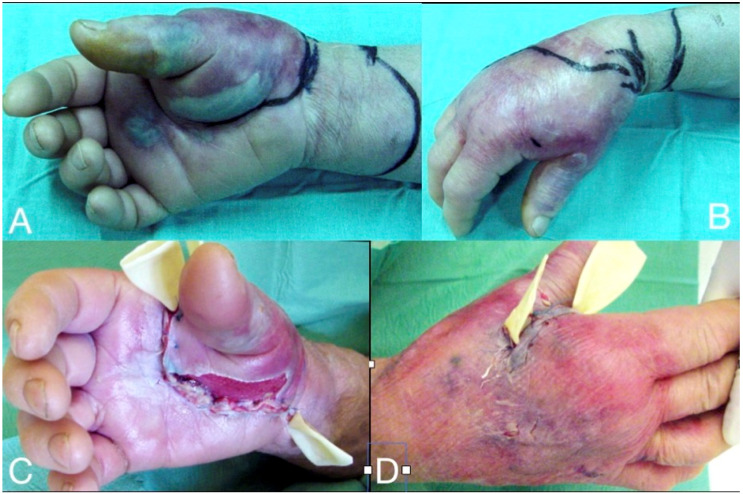
Deep infection at the thenar and the mid-palmar spaces, one week after a penetrating injury at the palmar aspect of the 1st phalanx of the thumb. The markings (**A**,**B**) demonstrate the margins of cellulitis at presentation, which improved significantly after administration of iv clindamycin and aminoglycoside for 24 h. Through a palmar incision, the palmar inter-muscular space of the thenar and the mid-palmar space were drained and debrided (**C**), followed by the dorsal thenar compartment debridement (**D**). The elastic tubes serve for the drainage until the wounds become dry.

**Figure 3:**
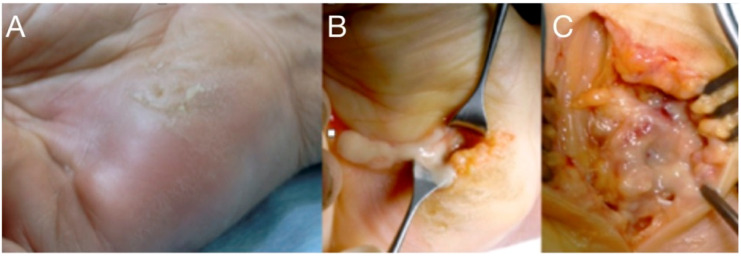
A neglected puncture wound at the ulnar side of the palm led to abscess formation into the hypo-thenar (**A**). Treatment included drainage, irrigation (**B**), and careful debridement protecting the ulnar neuromuscular bundle (**C**).

Certain anatomical and pathophysiological features are responsible for the specific clinical presentation of hand infections. The numerous mobile structures are lying superficially under the skin and in close proximity to the bones and joints. They are contained within a very limited space and surrounded by synovial membranes that behave as gliding surfaces interconnecting adjacent anatomical compartments. Thus, bacteria inoculated into deeper anatomical structures in case of penetrating wounds or open trauma and fractures, can be spread to contiguous anatomical spaces. The inoculated bacteria proliferate and disseminate to the adjacent tissue, causing micro-thrombosis, tissue necrosis, and may end up in abscess formation. When the septic material and exudates reach the synovial sheaths of mobile structures that serve as “pathways”, infection can be spread far and widely. The destruction of local anatomy further disseminates the infection into adjacent anatomical compartments and the deep spaces of the hand and forearm [4,5]. The infection may also spread through the lymphatic pathway to more proximal soft tissue compartments. The accumulation of purulent material increasing the pressure inside an anatomical compartment compromises the blood flow, causing ischemia and tissue necrosis; the infection is subsequently spread towards pathways of decreased resistance. Patients with asplenism, hepatic cirrhosis, diabetes mellitus, immune deficiency, and implants are at high risk of infection. The most frequent cause is poor initial care and delayed presentation [3,6]. An initially trivial distal finger infection, like felon or paronychia, if left untreated, might expand through the subcutaneous tissue towards the distal phalanx or to the distal inter-phalangeal joint and establish septic arthritis (Figure 1). They might even spread more proximally to the flexors or the extensors, causing septic tenosynovitis [6]. The anatomical differences between the palmar and dorsal structures may explain the different pathways of extension and the different clinical signs between these two sides of the hand when infected. The firmly anchored palmar skin to the underlined structures of connective tissue and to the palmar fascia hinders the spread of pus to the palm and orients it to the deeper palmar structures [6]. The skin of the dorsum of the hand has a mobile areolar subcutaneous layer, allowing the oedema to spread easily and give rise to extensive cellulitis. On the palmar aspect of the hand there are three potential closed spaces with well-defined anatomic boundaries, that are susceptible to infections: thenar, mid-palmar, and hypothenar spaces, located deeper to the flexor tendons, but superficially to the interosseous muscles. An initially superficial but expanding infection most commonly tracks and rapidly reaches the functionally important structures of the flexor compartments, including the mid-palmar space and the space of Parona through the carpal tunnel in the volar aspect of distal forearm. A similar pathogenesis characterizes abscess formation into the webs, the thenar, and hypothenar spaces [4,6,7] (Figure 2 and Figure 3).

## 3. Pathogens

Hand infections are usually attributed to the gram-positive cocci and especially to *Staphylococcus aureus* (most commonly) and the *Streptococcus β-haemolytic group A (pyogenes)*, followed by mixed organisms and gram-negative in immunocompromised patients. Gram-positive cocci range from 30 to 80% of positive cultures. This might be the result of skin flora inoculation during injury [5,8]. An emerging pathogen is the *Staphylococcus aureus* that carries the PVL gene; it is usually community-acquired and leads to necrotic lesions [8,9,10,11,12]. Hand injuries in fishermen and people working near to still water may be infected by *Mycobacterium marinum,* the identification of which, requires special conditions and culture means. Animal and human bites are a rather common cause of hand and forearm infections from a mouth flora, including anaerobes and gram-negative. Along with *Staphylococcus ssp.* (including *MRSA*) and *Streptococcus ssp.* (including *Streptococcus pyogenes),* the commonly isolated pathogens reportedly include *Pasteurella spp. (Pasteurella multocida, Pasteurella canis, Pasteurella dagmatis), Capnocytophaga canimorsus,* anaerobes (*Fusobacterium spp., Prevotella spp., Bacteroides spp., Porphyromonas spp.),* and others [13,14].

## 4. Clinical Appearance and Diagnosis 

Hand infections can lead to debilitating and often to permanent disability, particularly if they are not diagnosed early and treated promptly and properly. The unique anatomy of the hand, with its numerous enclosed spaces and compartments, warrants special considerations. Thorough history and examination are crucial in guiding further investigations and management, particularly because there are numerous mimickers of hand infections, such as gout and pseudogout, injection injuries, algodystrophy, metastases, and rheumatological disorders [4,7]. Prompt diagnosis and treatment are important, because hand stiffness, contractures, and even amputation or life-threatening complications can result from missed diagnoses or delayed treatment. 

**Figure 4:**
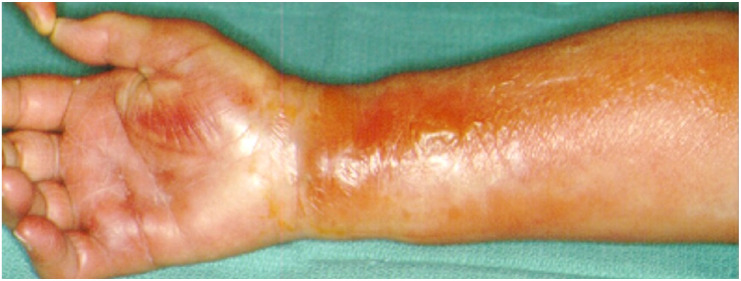
“Horse-shoe” abscess extended through the carpal tunnel, to the Parona’s space and the distal forearm.

**Figure 5:**
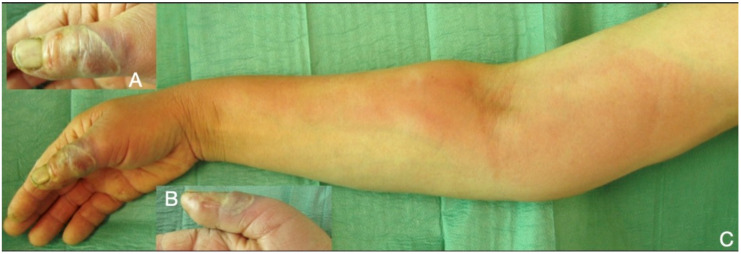
Necrotic cellulitis of the thumb (**A**,**B**), rapidly developed lymphangitis and spread till the lymph nodes of the distal humerus (**C**).

The knowledge of the closed-space anatomy of the hand and forearm, of the compartment syndrome pathophysiology, and the current updates in drainage/irrigation techniques and microbiology are prerequisites for prompt diagnosis and the optimal treatment of acute closed-space hand infections. (Figure 1A,B). The host’s general health status, the virulence of the microorganisms, comorbidity, immunosuppression, diabetes mellitus, and other metabolic abnormality will influence the severity of the clinical presentation [3,6,7]. The most common site of hand infections is subcutaneous tissue and the most common mechanism is trauma [5,8]. At an early stage, it might appear as a mild infection with typical cellulitis but without abscess formation. At a more advanced stage the infection is characterized by erythema, swelling, moderate cellulitis or erysipelas and lymphangitis, stiffness, a tense hand in intrinsic minus, and exquisite pain with passive stretch of fingers. Abscess formation will cause fever, malaise and debilitating pain (Figure 3a, Figure 4 and Figure 5). In the more severe hand infections, systemic symptoms with high fever, tachycardia, pain, and a white blood cell count above 12.000/μL, are common in immunocompromised patients with RA, diabetes mellitus, gout, peripheral vascular disease or renal failure, and in i.v. drug users, alcoholics, or patients under steroids [8,9,10,11,13,14,15,16,17,18,19]. This severe type might also occur when incision and drainage of an abscess followed by oral antibiotics, have failed. 

The ultrasound is the most convenient imaging examination for the hand infection. It is readily available, real time and easy to obtain, harmless, and low cost, without the need of sedation in children. Ultrasound imaging can help in differentiating a deep hand space infection from pyogenic flexor tenosynovitis or hematogenous spread infection from a distant site. It can also detect the presence of a foreign body into the soft tissue, the presence and the borders of an abscess, and the integrity of deep anatomical structures [20,21,22]. The MRI is the most sensitive in clearly depicting all of the different anatomical structures of the soft tissues, the joints, the bone and the bone marrow. 

When septic tissue, exudates, or aspirate samples are obtained, three sets of cultures should be ordered, each one including cultures for bacteria, mycobacteria, and fungi. Gram stain provides important information. While cultures are often false negative, recent advances in the detection and identification of bacterial pathogens by molecular methods greatly facilitate the diagnosis and expedite the initiation of treatment [18,23]. 

## 5. Therapeutic Approach

Penetrating wounds often cause acute hand infections and they are generally classified into superficial or deep infections. Superficial infections occur in the skin and subcutaneous tissues, whereas deep infections can involve the tendon sheaths, adjacent anatomic compartments, deep fascial planes, bursae, joint spaces, and bones. Deep fascial space infections are emergencies and warrant immediate surgical intervention. The pathway of inoculation, the environment where the initial injury occurred, and above all, the underlying condition of the host, must be taken into consideration for successful treatment. Superficial hand infections are more common and they are typically managed with rest, splinting in functional position, elevation, tetanus prophylaxis, analgesics, and empirical antibiotics. Anti-tetanus immunoglobulin plus tetanus toxoid vaccine, should be administered if the patient has not had a tetanus toxoid-containing vaccine in the past five years, especially in highly contaminated wounds [20]. 

The selection of empirical antibiotic treatment is mandatory, while waiting for the culture results. It depends on the origin of the contaminants, the local spread of community-acquired methicillin resistant *Staphylococcus aureus* (CA-MRSA), the type and severity of the infection, host factors, and local antibiotic resistance patterns. In crush injuries, injuries taking place in highly contaminated environment or in immunocompromised hosts, gram-negative bacteria and/or anaerobes are suspected. The route of administration is intravenous for all cases that require hospitalization, until the remission of the acute signs of infection. Subsequently, an effective oral regimen could be administered if available. Antibiotic treatment is usually initiated with penicillinase-resistant penicillin or cephalosporins. For more serious infections, the intravenous administration of vancomycin is recommended. Alternative intravenous therapies include daptomycin and aminoglycosides (amikacin). Not only penicillin and other β-lactams but also clindamycin and levofloxacin should be avoided during empirical antibiotic therapy, as methicillin resistant *Staphylococcus aureus* resistance to clindamycin and levofloxacin consistently increased during recent years. Antibiotics are usually required for seven to 10 days, unless complications arise. The oral empiric antibiotic treatment that is expected to be effective against suspected community-acquired methicillin resistant *Staphylococcus aureus* infections includes linezolid ciprofloxacin, clindamycin, rifampin, tetracyclines, and TMP (trimethoprim/sulfamethoxazole) [5,6,9,10].

If the infection is not subsiding, or an abscess is established, the treatment of choice is surgical management without delay. Hand infections spreading into deeper anatomical compartments are more severe and they require early surgical intervention and parenteral antibiotics. Missed diagnosis or delayed management will cause serious immediate morbidity and potential long-term disability, with a permanent loss of function. In the immune-compromised individuals, it might threaten limb viability and even life. The location of the infection, depth, and extent of the affected tissues are important for the selection of the appropriate surgical approach. The surgical incision and drainage of all potentially communicating spaces and compartments is mandatory, along with intra-operative irrigation and, sometimes, continuous postoperative irrigation. Specific approaches are proposed for the different locations of closed-space infections through safe anatomical paths (Figure 2 and Figure 3). In all cases, a bloodless field is imperative for the drainage and the evaluation of all potentially infected closed spaces. Special attention must be given to avoid the use of Esmarch’s bandage for exsanguination, so as to limit the spread of pus. Simple elevation of the hand and forearm is usually adequate for a bloodless field [6]. The persistence of acute surgical infections of the hand and wrist depends on patient and microbiology factors, as well as mechanism of bacteria inoculation and the depth of the tissue involved. Postoperatively, the hand is elevated and immobilized in a splint. There have been reports with risk-adjusted prognostic scoring system to anticipate which infections may require additional therapeutic debridement and they are also useful to counsel patients accordingly [24]. The treatment of hand infections is demanding, time-consuming, and not infrequently requires the infrastructure of a hospital and the skills of an experienced hand surgeon; therefore, early referral is always advisable in complex cases. Equally important is to initiate hand rehabilitation therapy sessions at the immediate postoperative period after the acute signs of infection subside.

## 6. Infections at the Deep Anatomical Compartments

The spread of an initially superficial infection from the site of inoculation to the deep structures might involve the tendon sheaths, adjacent anatomic compartments, deep fascial planes, bursae, joint spaces, and bones. Penetrating trauma often results in rapidly progressing severe infections within deep structures of the hand. The most common deep hand infections are: pyogenic flexor tenosynovitis, ulnar and radial bursitis, mid-palmar, thenar and hypothenar infections, horseshoe abscess, and infections that through the carpal tunnel spread in the space of Parona. Often, animal or human bites cause such infections (i.e., clenched fist injury) [5].

**Figure 6:**
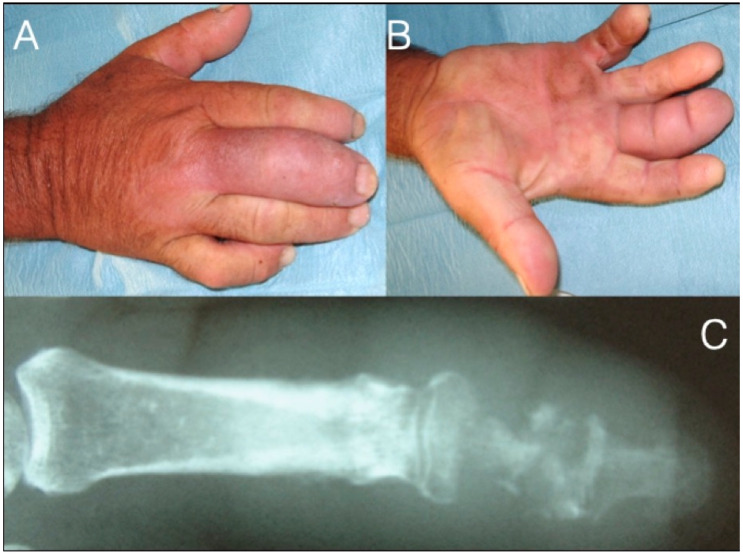
71-year-old farmer with a fusiform middle finger after penetrating injury 17 days ago through the dorsal skin just proximal to the DIP joint (**A**,**B**). He received analgesics and ampicillin per os in the last six days. The patient developed osteomyelitis of the middle phalanx and septic arthritis of the DIP joint (**C**). After debridement he underwent amputation at the PIP joint level.

Pyogenic flexor tenosynovitis is a closed-space infection of the flexor tendon synovial sheath that typically spans from the neck of the metacarpal to the distal inter-phalangeal joint with many anatomic variations [5]. A primary infection is commonly caused by direct inoculation from a penetrating injury to the finger and inoculation of the synovial fluid with bacteria [6]. The bacterial growth causes increased pressure within the sheath, leading to impaired vascular flow, severe impairment of the tendon’s gliding mechanism, tendon’s ischemia, and subsequent necrosis leading to tendon rupture. In cases of crushing injuries, infection comes from the direct contamination of the tendon sheath [23,25]. *Staphylococcus species*, including methicillin-resistant *Staphylococcus aureus*, and *Streptococcus species,* are the commonly identified pathogens. Approximately one-third of wound cultures show no growth, while gram-negative organisms are present in 10% of cases. Haematogenous spread to the tendon sheaths is less common and mainly occurs in immunocompromised patients [19,25,26]. Secondary infections are carried either through the lymphatics or through pus spreading from adjacent fascial spaces [6]. Tobacco use is an identified risk factor for more serious infections and delayed wound healing [23,25]. The diagnosis lacks laboratory or radiologic signs, except the ultrasound, and it is clinically based on the useful tool of the four Kanavel’s clinical signs, including: fusiform swelling of the finger, pain on passive extension, a partially flexed resting posture of the finger, and volar tenderness along the length of the finger and into the palm. No other clinical examination has proven to be superior to date (Figure 1 and Figure 6). However, flexor tenosynovitis can be initially present without all four Kanavel’s signs and caution should be applied when using the absence of one or more Kanavel’s signs to exclude the diagnosis [27,28].

Early pyogenic flexor tenosynovitis can be non-operatively managed, although no specific protocol exists. If there is no improvement by systemic administration of antibiotics within 48–72 h from symptom onset, a minimal incision with catheter irrigation is the mainstay of surgical treatment and results in an improved range of motion and fewer infectious complications compared with open surgery [22]. In more severe infections, drainage through a wide or limited approach, in a bloodless field, is necessary. The wide approach is either lateral or palmar (zigzag), whereas the limited consists of two small incisions over the two edges (A1 and A5 pulleys) of the infected sheath. Surgeons advocating the limited approach claim a better final range of motion, while others believe that a limited approach should be used in less severe cases, although there are no level I studies comparing the type of incision. Giladi and colleagues in 2015, advocate that intra-operative and postoperative closed sheath catheter irrigation with normal saline for 2–3 days, until the resolution of the acute inflammation, achieved improved range of motion when compared with open washout [6,22,24,28,29,30,31].

**Figure 7:**
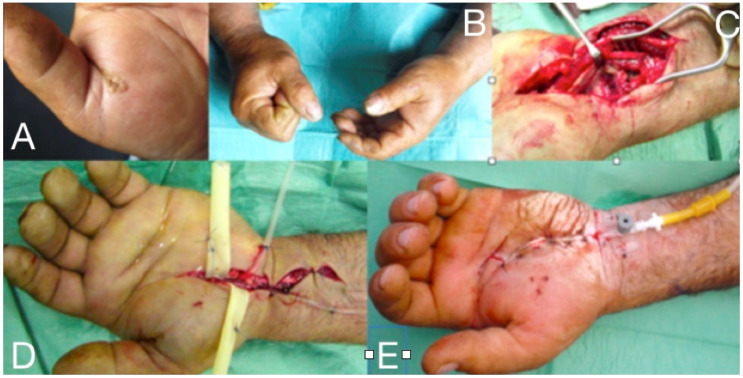
Thenar space infection due to penetrating wound in the palmar surface (**A**). Infection is extended to distal forearm (**B**). Thenar space, carpal tunnel and Parona’s space were incised, drained and thoroughly irrigated while median nerve is recognized and protected (**C**,**D**). Continuous postoperative irrigation can be applied without preventing early postoperative mobilization (**E**).

The synovial sheaths of both the flexor polices longus continues proximally as radial bursa, whereas the tendon sheaths of the ulnar digits or only the little fingers, continue proximally as the ulnar bursa, where both communicate with each other via the carpal tunnel to the space of Parona. This is a potential space at the level of the distal forearm, between the profundus tendons and the pronator quadratus [27,29,32] (Figure 7). Ulnar bursitis of the hand is characterized by the development of hand oedema, especially upon the dorsal aspect, and it is often difficult to be diagnosed due to its deep location. A general fullness is seen in the palm, but the palmar concavity is not lost at first. There is exquisite tenderness and the wrist becomes fixed, whereas the little finger and sometimes ring finger show tenderness to palpation and pain on passive extension. Extension to the radial bursa is observed up to 85% of cases [27,28]. Radial bursitis is diagnosed by the swelling and tenderness in the thenar eminence and along the radial bursa [28]. An infection that starts in the thumb might spread to the fifth digit via the wrist and vice-versa due to this connection, thus creating a horseshoe shaped abscess [27] (Figure 4). Special attention should be paid when tenderness is abruptly subsiding. It does not necessarily mean a definite improvement, as it might only be a temporary relief due to the rupture of the infected sheath at its proximal edge and, therefore, of extension of the pus to more proximal structures. Differential diagnosis should include acute bleeding into the tendon sheath in patients under anticoagulation therapy and also tenosynovitis developing in 2/3 of patients with gonococcal infection [32]. The treatment in cases of purulent accumulation consists of surgical drainage, debridement of abscesses through a palmar incision over the infected area, followed by intra-operative irrigation with normal saline. Care should be taken not to injure the branch of the median nerve, which supplies the thenar muscles as it passes across the radial bursa, approximately 1 cm distal to the transverse ligament of the wrist. In both tenosynovitis and bursitis, surgical wounds can be closed by secondary intention unless there is continuous postoperative irrigation. Passive assisted and active exercises to restore the range of motion, start with the remission of acute inflammation, and, even if the irrigation system is in place or after the removal of the irrigation system [28,30,31,33,34,35,36,37,38] (Figure 7).

Francesco Parona was an Italian surgeon who first described the space between the pronator quadrates and the flexor tendons in 1876 [39]. Infection of the Parona’s space is a rare and potentially limb threatening complication and it must be suspected in patients with flexor tendon sheath infections of the thumb or little finger. Early antimicrobial therapy directed particularly at *β-haemolytic Streptococcus* combined with prompt surgical debridement and physiotherapy are critical for an optimal functional outcome [28]. Deep space abscesses involving the thenar, the mid-palmar, the Parona’s space, or the interdigital web spaces of the hand usually arise from direct inoculation or contiguous spread (Figure 7). Marked swelling, oedema, warmth, and pain are prominent, and the ultrasonography or magnetic resonance imaging may help in the differentiation from a superficial infection [27,29,32].

Hand and forearm infections from animal or human bite injuries are common. A bite might initially appear trivial and harmless, but might involve clinically significant tissue injury in the depths of the wound. Bites can transmit unusual pathogens of the saliva into the wound. The risk of infection after a bite is about 20–40%, and approximately 20% of dog bites will become infected in children [40,41]. Prophylactic antibiotics selected according to the nature of the wound, the location, the oral flora bacteria species of the biting animal, and the characteristics of the patient, but are only recommended for wounds that are considered at high risk of infection. Those might be the deep or contaminated wounds with extensive tissue destruction and poor perfusion and those involving bones, joints, and tendons [41,42,43,44]. About 30–60% are due to mixed aerobic-anaerobic pathogens. The index finger is the most common site of injury in cat bites (45%), with 40% of cases causing flexor tendon sheath infection of the fingers [44,45]. *Pasteurella species* are isolated from 35% of the infected wounds. Human bites often require hospital admission, repeated debridement and irrigation, antibiotics, and delayed closure [45,46,47,48]. The most commonly isolated pathogen in human bite wounds is *Eikenella corrodens.* Aside from wound infections, bites can also cause systemic bacterial infection [45,49,50,51,52,53,54].

Clenched fist injury is a 3–5 mm laceration over the metacarpophalangeal joint or proximal interphalangeal joint, being often sustained from incisor teeth touching to the bone or into the joint. After the extension of the fingers the tendon slides backwards covering the lacerated joint capsule where the contaminants from the teeth grow and induce an infection, which, if neglected, might aggravate and expand towards the dorsal sub-aponeurotic space of the dorsal hand and cause septic arthritis of the adjacent joint [55,56]. Hospitalization and consultation of a hand surgeon are necessary for all bite wounds of the hand that involve the bones and/or joints. Particular care must be undertaken for tetanus prophylaxis. Full vaccination should be provided if there is any doubt whether the patient is adequately immunized against tetanus [29,32]. Prophylaxis is rarely necessary for the rabies potential exposure from any scratch or bite wound by an animal that may be infected, or from contact of such an animal’s saliva with a human’s mucous membrane. It is given after evaluation of the risk and in coordination with the local veterinary authorities, the nature of the contact, the species of the animal, and the current rabies situation in the local geographical area [56,57,58,59]. Local treatment requires thorough wound debridement of devitalized tissue, cleansing with 1% povidone-iodine solution, and irrigation with normal saline. Surgical debridement is superior to irrigation alone, but might be required in multiple operations secondary to septic arthritis, osteomyelitis, necrotizing fasciitis, and deep collection. Primary wound closure or healing by second intention depend upon the adequacy of debridement. Tissue cultures are indispensable; intravenous empirical antibiotics should be used and tailored postoperatively according to susceptibility tests in the hospital, continued with outpatient follow-up. The limb should be immobilised and initiate hand therapy, as tolerated [27,60,61].

**Figure 8:**
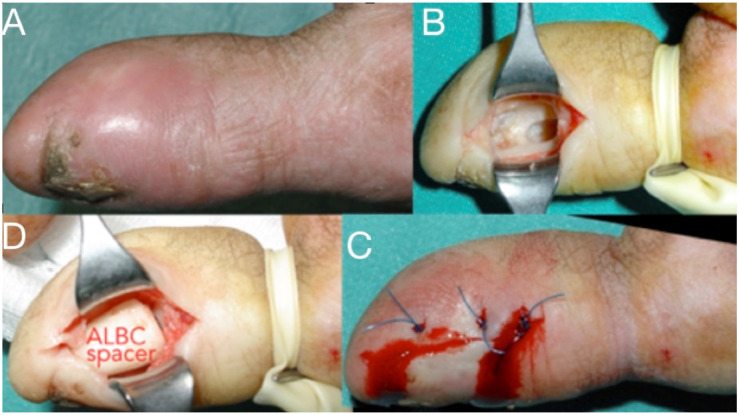
Infection at the distal phalanx of the thumb after a crash injury left for secondary healing (**A**), Thorough debridement from a lateral approach (**B**), antibiotic-loaded bone cement spacer for three weeks and wound closure (**C**,**D**). The spacer will be replaced with bone graft at a second stage.

Septic arthritis typically results from penetrating injuries or contiguous spread into the joint space, causing swelling, painful, and restricted range of passive and active motion [61,62]. The joint aspiration reveals more than 50,000 white blood cells (more than 75% are polymorphonuclear lymphocytes) [62,63,64,65,66]. Radiographs demonstrate joint space narrowing and bone erosions. If neglected, it extends into the bone causing osteomyelitis. Prompt diagnosis and surgical debridement with synovectomy and culture-guided i.v. antibiotics (intravenous and/or oral) for as long as the local signs subside is the treatment of choice [67,68]. Antibiotic-loaded bone cement spacers are also used (Figure 1 and Figure 8). In children with acute osteomyelitis, a short course of intravenous antibiotics can be followed by oral administration (only if equally effective oral alternatives exist) [69,70,71,72,73]. Primary osteomyelitis of the hand is uncommon, but, if not adequately and promptly treated, the detrimental effects on hand and wrist function can be devastating [67,68,69]. Radiographic changes may include osteopenia, periosteal reaction, and lytic areas [66,67,68,69,70,74].

Necrotizing soft tissue infections (NSTI) of the hand and the upper limb include necrotizing fasciitis, which is the most common, followed by myonecrosis and necrotizing cellulitis [75,76,77]. They can either be mono-microbial, caused by a single bacterial species, or poly-microbial, caused by diverse microorganisms. *Streptococci species* (as *Streptococcus pyogenes, Streptococcus dysgalactiae, Streptococcus agalactiae), Staphylococcus aureus* and *MRSA* are the most common pathogens reported to cause mono-microbial NSTIs. Polymicrobial NSTIs are associated with a mixture of aerobic and anaerobic bacteria, including *Enterobacteriaceae, Bacteroides spp., Porphyromonas spp., Prevotella spp., Peptostreptococcus spp.*, and *Clostridium spp* [76,77,78]. The bacteria causing necrotizing infections can spread rapidly, causing an acute, and rapidly progressive, clinical appearance along the skin, the subcutaneous tissue, and the fascial planes, which results in cellulitis, swelling, tenderness, and erythema of the skin surrounding the affected area, with unregularly marginated edges. The skin is warm to the touch, very painful, especially in the early stages. Within 2–3 days from the onset, blisters start to emerge, rapidly evolving into skin necrosis (Figure 2, Figure 4 and Figure 5). The incidence of systemic signs, including fever, tachycardia and severe hypotension, dizziness, fatigue, diarrhea, or nausea is high [76,77,78,79]. The poly-microbial NF is commonly observed in individuals with underlying comorbidity, diabetes, or older patients, but the mono-microbial ones are more commonly associated with trauma, surgery, or intravenous drug use. *Vibrio vulnificus* should be considered as a possible cause of necrotizing fasciitis in wounds that were exposed to coastal waters. Treatment should be promptly initiated with resuscitation measures, i.v. doxycycline and a third generation cephalosporin or fluoroquinolone, and aggressive debridement, fasciotomy, or even amputation may sometimes be necessary to improve survival [79,80,81]. The pus has a characteristic “dishwasher” odor. Blood and affected tissue or hemorrhagic bullae liquid cultures are recommended. The mortality varies from 20–45% and the reported amputation rates are high. Early diagnosis might be challenging and appears to be crucial in management. Surgical debridement of NSTIs within 12 h is essential for reducing the mortality rate, while surgical treatment within 6 h might further improve outcomes [80,81,82,83].
